# Early and Late Postoperative Atrial Fibrillation After Coronary Artery Bypass Grafting and Surgical Aortic Valve Replacement: An Exploratory Study on a Dual-Modality Ambulatory Electrocardiogram Monitoring

**DOI:** 10.3390/diagnostics16050670

**Published:** 2026-02-26

**Authors:** Andrzej Kułach, Tomasz Skowerski, Magdalena Piekarska, Michał Majewski, Marek Deja, Wojciech Wańha, Radosław Gocoł, Zbigniew Gąsior, Grzegorz Smolka

**Affiliations:** 1 Department of Cardiology, School of Health Sciences, Medical University of Silesia, 40-635 Katowice, Poland; 2 Department of Cardiac Surgery, School of Health Sciences, Medical University of Silesia, 40-635 Katowice, Poland; 3 3rd Department of Cardiology, School of Medicine, Medical University of Silesia, 40-635 Katowice, Poland

**Keywords:** postoperative atrial fibrillation, coronary artery bypass grafting, aortic valve replacement, Holter patch, event recorder

## Abstract

**Background:** Postoperative atrial fibrillation (POAF) after cardiac surgery is common and clinically relevant, yet optimal postdischarge ECG surveillance remains undefined. We assessed the incidence of POAF after isolated coronary artery bypass grafting (CABG) and surgical aortic valve replacement (SAVR) using a dual-modality ambulatory strategy. **Methods:** In an exploratory, single-center study, consecutive adults without pre-operative AF undergoing elective isolated CABG or SAVR received dual-modality monitoring after discharge: continuous patch-Holter for ~10 days and a patient-activated single-lead recorder for up to 30 days. Early POAF was AF/AFl during index hospitalization; late POAF was first AF/AFL detected postdischarge by either modality. **Results:** Fifty-five patients were enrolled (CABG 30 [54.5%], SAVR 25 [45.5%]; mean age 64.6 ± 9.8 years; 38.2% women). Early POAF occurred in 10/49 (20.4%); late POAF was detected in 21/55 (38.2%). By modality, late AF was identified on the 10-day Holter in 11/51 (21.6%) and on the 30-day recorder in 19/51 (37.3%). Cumulative detection reached 20.0% by day 7, 30.9% by day 10, and 38.2% thereafter, demonstrating that a substantive proportion of late POAF occurred after day 10, and 19/21 (90%) were captured by event monitoring. Female sex was independently associated with late POAF (OR 3.70, 95% CI 1.17–11.72); longer aortic cross-clamp time was related to late POAF in the SAVR subset, while larger LA size was related to POAF incidence in the CABG group. Early (in-hospital) POAF was associated with subsequent late POAF (*p* = 0.025). The difference in late POAF frequency between CABG and SAVR (33.3% vs. 44.0%; *p* = 0.42) was not significant. **Conclusions:** Among patients without prior AF undergoing CABG or SAVR, late POAF is frequent and often manifests beyond 10 days after discharge. Extending ambulatory surveillance to 30 days—or adopting a 10-day continuous plus patient-activated to day 30 hybrid—materially improves case finding and should be considered in routine postoperative pathways.

## 1. Introduction

Postoperative atrial fibrillation (POAF) is defined as new-onset atrial fibrillation (AF) occurring after cardiac surgery in patients without a prior history of AF. It typically arises within the first postoperative week, most commonly between days 2 and 3, and may recur intermittently for weeks thereafter. The 2024 and 2020 European Society of Cardiology (ESC) AF Guidelines classify this arrhythmia under “trigger-induced AF” and “post-operative AF”, acknowledging that its onset is precipitated by the acute surgical and inflammatory milieu rather than an established atrial substrate [1,2].

Early postoperative atrial fibrillation (early POAF) is defined as any new-onset atrial fibrillation or atrial flutter episode occurring during the index hospitalization and detected by intensive care or ward monitoring (telemetry, 12-lead ECG, or continuous ECG recording), irrespective of episode duration. Late POAF is defined as atrial fibrillation/atrial flutter first detected after hospital discharge (commonly up to 30 days), typically by ambulatory or opportunistic ECG monitoring.

The pathophysiology of POAF reflects the interaction of transient triggers—systemic inflammation, catecholamine surge, volume shifts, and pericardial irritation—with pre-existing atrial vulnerability (age-related fibrosis, atrial stretch, or diastolic dysfunction). Studies in surgical series demonstrate that atrial dilation, oxidative stress, and autonomic imbalance act synergistically to lower the threshold for atrial re-entry, confirming that AF promotes more AF even in the postoperative setting [3].

### 1.1. Epidemiology and Prevalence

POAF remains the most frequent arrhythmic complication after cardiac surgery, occurring in 20–30% of patients following coronary artery bypass grafting (CABG) and up to 40% after surgical aortic valve replacement (SAVR). Incidence peaks within the first 72 h and declines to baseline by six weeks [4]. Risk factors include older age, atrial enlargement, heart failure, and perioperative inflammation, which is linked to AF but also to ectopy burden [5]. In general thoracic procedures, AF occurs in 12–44% of patients, underscoring the systemic and pericardial contribution to arrhythmogenesis [3].

Historically regarded as a benign, self-limited postoperative event, POAF is now recognised as a marker of adverse outcomes. The 2021 meta-analysis by Eikelboom et al., comprising more than 155,000 cardiac surgery patients, demonstrated that POAF is related to higher mortality and a higher risk of stroke and permanent AF extending up to 10 years postsurgery [4]. Another systematic review of 179 studies (*N* = 241,712) analizing new onset of atrial fibrillation after TAVI, show that AF occurred in ~9.9% of patients and was linked to early (≤30-day) stroke (RR 2.35) and higher 30-day mortality (RR 1.76), reinforcing that postprocedural AF, even outside surgery, is not benign [6]. In SAVR-specific cohorts, new-onset POAF associates with long-term death and stroke; in 7038 isolated SAVR and 3854 SAVR + CABG patients from Swedish nationwide registries, POAF conferred adjusted hazards for death (aHR 1.21 and 1.31, respectively) and ischemic stroke in isolated SAVR (aHR 1.32), while also predicting HF hospitalization and recurrent AF on follow-up [7].

These findings challenge the concept of transient, harmless post-op arrhythmia and suggest that early postoperative AF may unmask latent atrial disease with implications for long-term rhythm and stroke prevention strategies.

### 1.2. Prevention and Treatment Strategies

Peri- and postoperative prophylaxis remains focused on modulation of sympathetic tone and inflammation. The 2024 ESC guidelines, along with the Society of Thoracic Surgeons (STS) guidelines, recommend continuation of preoperative β-blockers (Class I, Level B) and consider amiodarone prophylaxis as reasonable alternatives (Class IIa, Level B).

In established POAF, management is similar to that in nonsurgical AF care: β-blockers, pharmacological, or electrical cardioversion for hemodynamic instability and individualized anticoagulation depending on AF duration and thromboembolic risk [3].

Both 2024 ESC AF guidelines and ACC/AHA 2023 AF guidelines regard POAF as secondary or trigger-induced AF. Key recommendations regarding ECG monitoring and anticoagulation treatment after surgery include:

- Structured postdischarge rhythm surveillance and reassessment of stroke risk, as many patients develop recurrent or subclinical AF months after surgery.
- Consideration of long-term oral anticoagulation in patients with elevated CHA_2_DS_2_-VASC/VASc scores, even if sinus rhythm is restored, acknowledging persistent thromboembolic risk [1,2,8].

However, guidelines highlight major evidence gaps: lack of consensus on AF duration thresholds warranting anticoagulation, insufficient data on the clinical significance of late or paroxysmal POAF detected by ambulatory monitoring, and uncertainty regarding the long-term benefit of aggressive rhythm control after surgery.

### 1.3. Complications and Long-Term Sequelae

POAF increases stroke, heart failure, prolonged hospitalization, and perioperative mortality, with added healthcare costs and reduced quality of life. Late complications include recurrence of AF in up to 25–30% of cases within five years, stroke risk equivalent to non-surgical AF, and progression to structural atrial remodeling. Left atrial appendage thrombus formation remains a major concern, particularly in patients discharged in sinus rhythm without sustained monitoring.

The 2024 ESC guideline explicitly recommends extended ECG follow-up and re-evaluation of anticoagulation at three to six months in patients with postoperative or trigger-induced AF, reflecting the paradigm shift from acute arrhythmia management to chronic risk surveillance [3,4,8,9,10].

### 1.4. Rationale for the Study

Despite advances in perioperative prophylaxis and evolving guideline frameworks, the temporal pattern and prognostic relevance of early versus late POAF remain poorly described. Conventional in-hospital ECG monitoring underestimates the arrhythmic burden, while ambulatory ECG modalities enable the detection of asymptomatic or delayed episodes. Patient-activated, handheld, single-lead ECG event recorders have been proposed as useful tools in symptomatic patients (dizziness, palpitations) and, in some clinical settings, are more effective than conventional 24 h Holter-ECG [8]. Due to their low cost and good specificity in arrhythmia detection, they are being used in many conditions where a proper diagnosis is crucial for further treatment. In this exploratory study, performed before planned large-scale telemetry surveillance in post cardiac surgery subjects, we assess dual-modality ambulatory ECG monitoring, combining 10-day continuous ECG surveillance with patient-activated event recording for 30 days after discharge to seek late POAF after CABG and SAVR, quantifying true arrhythmic burden (asymptomatic events in early postdischarge period) and symptom-related findings throughout the monitored time of 30 days.

## 2. Methods

We performed an exploratory, prospective, single-center study in two surgical cohorts—isolated coronary artery bypass grafting (CABG) and isolated surgical aortic valve replacement (SAVR)—to compare in-hospital (early) and postdischarge (late) postoperative atrial fibrillation (POAF) using a dual-modality ambulatory ECG strategy (continuous Holter patch and patient-activated event recorder). The protocol, patient information sheet, and consent form were approved by the Bioethics Committee of the Medical University of Silesia, Katowice, Poland (KNW/0022/KB1/57/16; Date: 6 June 2016) and conformed to the Declaration of Helsinki. All participants provided written informed consent. Patients or the public were not involved in the design, conduct, reporting, or dissemination plans of our research.

### 2.1. Participants

#### 2.1.1. Eligibility and Enrolment

Consecutive adults undergoing elective isolated CABG or isolated SAVR were screened. We excluded patients with (i) known atrial fibrillation/flutter, (ii) severe left ventricular systolic dysfunction (LVEF < 40%), and (iii) other conditions precluding safe monitoring or informed consent. A total of 55 patients were enrolled: 30 after CABG and 25 after SAVR.

#### 2.1.2. Baseline Variables and Imaging

Demographic and clinical data included age, sex, height/weight (BMI), hypertension, diabetes, prior myocardial infarction, and peripheral/vascular disease, along with symptom status and functional class as available. Risk scores were calculated as CHA_2_DS_2_-VA and HAS-BLED. Transthoracic echocardiography was performed pre-operatively; recorded parameters included LVEF, mitral regurgitation grade, left atrial diameter (LA), and left atrial volume (LAV).

#### 2.1.3. Surgical/ICU Variables and Early POAF

Operative variables (CABG: number of grafts, arterial graft use, on- vs. off-pump; SAVR: bioprosthesis vs. mechanical valve) and early postoperative/ICU variables (ICU stay, vasopressor time, early POAF occurrence, complete blood count, renal function, potassium) were abstracted from charts into the study database prior to ECG analysis. Cross-clamp time and on/off-pump status were available in subsets. Early POAF was defined as any AF/flutter episode documented in-hospital (ICU/ward telemetry or Holter during index hospitalisation), lasting at least 30 s. Termination mode (spontaneous, amiodarone, electrical cardioversion) was recorded.

### 2.2. Postdischarge Rhythm-Monitoring Protocol

#### Dual-Modality Strategy and Timing

To capture both asymptomatic and symptomatic AF after discharge, we used:

1. Continuous Holter patch for ~10 days;
2. Patient-activated handheld single-lead event recorder for 28 ± 2 days, with twice-daily 30-s recordings plus symptom-triggered strips.

This schedule was initiated immediately postdischarge or as soon as logistically feasible after ward transfer, with actual recording lengths verified in device logs. (Holter median 9 days). Ambulatory monitoring used a Bittium Faros 3-channel patch recorder (Bittium, Oulu, Finland) with Omega Snap electrodes; analyses were performed in Bittium Cardiac Navigator (version 1.6). A handheld, patient-activated, single-lead device CheckMe Pro, (Viatom Technology Co., Ltd., Shenzhen, China) was used for event capture for 28 days. The schedule comprised 10 days of continuous Holter patch combined with 28 days of patient-activated intermittent recordings, with all tracings adjudicated by a cardiologist.

### 2.3. Device Setup and Patient Instruction

Technicians placed the 3-lead patch before discharge with standard skin prep; patients were instructed to keep the patch in place for 10 days (or until electrode replacement per skin tolerance). For the event recorder, participants were trained to record two routine strips daily (morning/evening) and additional strips for palpitations, dizziness, dyspnoea, chest discomfort, or syncope. They received an instruction card with common artefacts and positioning tips to maximize signal quality, consistent with prior group practice.

### 2.4. ECG Data Handling and Adjudication

Holter data were analysed by a cardiologist in Bittium Cardiac Navigator; outputs included minimal/average/maximal HR, supraventricular tachycardia (SVT) runs, AF episodes (with day of occurrence, episode duration, and maximal HR), bradycardia (<50 bpm), and AV-conduction disorders. Event recorder strips were adjudicated manually; parameters logged included minimal/maximal HR on strips, day of monitoring, number of AF-positive strips, and max HR during AF. All recordings were analysed by a board-certified cardiologist. All recordings containing clinically relevant arrhythmic events, including atrial fibrillation, were independently reviewed by a second cardiologist. In case of discrepant interpretations, the recordings were adjudicated by a senior electrophysiologist, whose assessment was considered final.

### 2.5. Definitions of Endpoints

- Late POAF (primary late endpoint): any postdischarge AF/flutter episode detected by either modality (Holter patch or event recorder), lasting at least 30 s, during the 30-day ambulatory phase.
- Modality-specific POAF detection: late AF detected on Holter only, event recorder only, both, or neither were used for paired comparisons of diagnostic yield (see Statistics).

### 2.6. Sample Size and Data Completeness

This was an exploratory cohort with a target sample of ~50–60 patients (achieved *N* = 55), adequate for estimating modality-specific detection proportions and running a limited multivariable model. Two cardiologists independently adjudicated ambiguous AF strips; disagreements were resolved by a senior electrophysiologist. Device logs and strip counts were cross-checked against patient diaries to confirm adherence.

### 2.7. Statistical Analysis

All analyses were pre-specified and conducted in MedCalc (v22.026/23.2.6). Continuous variables were presented as mean ± SD if normally distributed and as median with IQR otherwise; normality was assessed using the Shapiro–Wilk test. Group comparisons used Student’s *t*-test (normal distribution) or Mann–Whitney U/Wilcoxon (non-normal distribution) for continuous variables, and Pearson’s chi^2^ or McNemar’s test for categorical/paired proportions as appropriate. Spearman’s rank correlation tested associations of non-normal metrics.

The logistic regression analysis was performed to determine independent predictors of endpoints.

A two-sided *p* < 0.05 defined statistical significance.

To directly compare the diagnostic yield of late POAF between the two ambulatory strategies within the same subjects, we used a paired McNemar test on the 2 × 2 table of detections (Holter only vs. event-recorder only vs. both vs. neither).

### 2.8. Ethical and Safety Considerations

Per standard of care, clinically significant arrhythmias detected during monitoring were communicated to treating teams for management (rate/rhythm control, anticoagulation). The non-invasive monitors carry minimal risk; skin irritation was managed with electrode changes as needed. No device-related serious adverse events were recorded.

## 3. Results

### 3.1. Study Population and Baseline Characteristics

Fifty-five patients were enrolled (CABG *n* = 30 [54.5%], SAVR *n* = 25 [45.5%]); 21 (38.2%) were women. Mean age was 64.6 ± 9.8 years (median 67). Hypertension was present in 36/55 (65.5%), diabetes in 13/55 (23.6%), and prior myocardial infarction in 12/55 (21.8%). Stroke history was absent (0/55). Median CHA_2_DS_2_-VA was 2 (IQR 0–3) and HAS-BLED 1 (IQR 0–1). Pre-operative echocardiography showed median LVEF 55% (IQR 50–60%), left atrial diameter 41 mm (IQR 38–44), and left-atrial volume ~23 mL (IQR 22–27). SAVR prostheses were predominantly bioprosthetic (19/25, 76%). In CABG patients with available data, the number of distal anastomoses was 2–3 in 20/21 (95%). One patient died of stroke 8 days after discharge with an AF documented patch recorded (starting day 4) and in the event monitor recordings. One more patient died of an unknown reason within the FU period.

Detailed baseline distributions are provided in Table 1.

### 3.2. Monitoring Exposure and Adherence

Continuous Holter patch was worn for a median of 9.0 days (IQR 8.0–10.9). The patient-activated recorder was used for a median of 21 days (IQR 13–28) with a median of 39 strips (IQR 22–50) recorded per participant. Adherence rates for Holter patch was 93% (monitoring returned <3 days in 4 cases) and for event monitoring: 93% (4 cases never used the device). The analyzable ECG time in the Holter patch was >95% in all cases except one, in which, despite artefacts, a >24 h episode of AF was recorded. Patient-activated handheld ECG recorders were used properly with no apparent operational issues. The total number of strips was 1888, and 1810 (96%) of ECG strips were analysable. Detailed monitoring data are provided in Table 2.

### 3.3. Early and Late POAF Detection

Early POAF occurred in 10/55 (18.2%) and in all cases was captured by a post-op or ward telemetry. In all cases, iv amiodarone was used; three cases required electrical cardioversion.

Postdischarge AF (late POAF documented by any modality) was detected in 21/55 (38.2%). By modality, late AF was identified on the 10-day Holter in 11/51 (21.6%) and on the 30-day patient-monitor in 19/51 (37.3%). The median AF episode duration assessed in the Holter patch was median 37 h (IQR: 10–102 h). Except for one case which lasted 24 min, all episodes lasted more than 8 h.

There was no significant difference in late AF detection between CABG and SAVR (CABG 33.3% vs. SAVR 44.0%; χ^2^ *p* = 0.42).

### 3.4. Head-to-Head Modality Comparison

In a paired, within-patient analysis using McNemar’s framework, atrial fibrillation was detected by both modalities in 9 patients, by Holter-only in 2, by the 30-day patient-activated monitor PM-only in 10, and by neither modality in 34 (*n* = 55). Atrial fibrillation detection was concordant in 43 patients (9 positive by both modalities and 34 negative by both). Discordant results occurred in 12 patients, with AF detected by the 30-day patient-activated monitor in 10 patients and by Holter only in 2 patients. The excess of PM-only episode over Holter-only detections is obviously a result of the Holter monitoring duration, but it indicates a higher diagnostic yield of the 30-day strategy for late POAF.

In time-to-event analysis of first postdischarge AF detection, cumulative yield reached 20.0% by day 7, 30.9% by day 10, 32.7% by day 14, and 38.2% by day 30 (overall 38.2%, *n* = 55).

### 3.5. Predictors of Late POAF

On univariable analyses, female sex was associated with late POAF (χ^2^ 5.08, *p* = 0.024), whereas hypertension and diabetes were not (both *p* > 0.5). In a logistic regression model, female sex remained independently associated with late POAF (OR 3.70, 95% CI 1.17–11.72; *p* = 0.026). Procedure type (CABG vs. SAVR) was not significant.

Among operative/ICU variables, longer aortic cross-clamp time in the SAVR group was observed in patients with late AF (median 66.0 vs. 46.5 min; *p* = 0.004), while ICU stay and vasopressor time did not differ significantly.

LA size showed a borderline independent association with late POAF in the CABG group (OR 1.32 per 1 mm, 95% CI 0.97–1.79; *p* = 0.075)

Of note, given the limited sample size, both univariable and multivariable analyses should be interpreted as hypothesis-generating, irrespective of statistical significance.

### 3.6. Relationship Between Early and Late POAF

Patients who experienced early (in-hospital) POAF were more likely to manifest late POAF after discharge (χ^2^ 5.06; *p* = 0.025).

### 3.7. Differences in Clinical Parameters and Rhythm Disturbances by Late POAF Status

In this cohort, late POAF was not associated with differences in age, hypertension, or diabetes. Female sex trended higher in the late-POAF group (47.6% vs. 26.5%; *p* = 0.147). The data are presented in Table 3.

## 4. Discussion

In this prospective cohort of CABG/SAVR patients, dual-modality ambulatory monitoring (10-day continuous Holter patch plus 30-day patient-activated recorder) identified late POAF in 38.2% overall, with PM-only detections exceeding Holter-only detections (10 vs. 2) on within-patient comparison, indicating a higher incremental diagnostic yield of the 30-day strategy for postdischarge case finding. These data align with contemporary evidence that POAF is frequent and prognostically relevant, challenging the notion that it is transient or benign [4].

Large observational studies show that POAF is associated with higher long-term mortality and stroke beyond the index hospitalization. In a meta-analysis of >150,000 patients, POAF increased 1-year death (OR ≈ 2.6) and long-term mortality (adjusted HR ≈ 1.25). Also in a PARTNER-3 trial, early POAF (in-hospital) did not independently predict the 2-year composite of death/stroke/rehospitalization, whereas late POAF did (adjusted HR 8.90), irrespective of treatment modality [11]. Our finding that a substantial share of first detections accrued after day 10 supports extended surveillance to uncover clinically meaningful AF otherwise missed by short monitoring [4].

Major AF guidelines now classify AF in the setting of surgery as trigger-induced/secondary AF and recommend structured postdischarge reassessment, but there are no clear recommendations on the duration and modality of monitoring for cardiac-surgery patients. The 2024 ESC AF guidelines place POAF under “trigger-induced AF”, advise management per AF-CARE principles, and explicitly lists improved stroke risk stratification for postoperative AF as an evidence gap. The guidelines do not provide a standard for postdischarge ECG surveillance (e.g., 10 vs. 30 days, continuous vs. intermittent). The 2023 ACC/AHA/ACCP/HRS AF guidelines likewise highlight that AF first detected during acute illness or surgery warrants postdischarge follow-up, risk stratification, and AF surveillance, without specifying a concrete monitoring pathway for POAF cohorts. Our data address a part of this gap by demonstrating incremental yield beyond 10 days with a patient-activated modality [1,2,7].

CABG/SAVR populations typically carry elevated CHA_2_DS_2_-VA scores, and multiple studies have shown higher scores in those who develop POAF (often medians around 3 in POAF vs. 2 without POAF). Observational work also links higher CHA_2_DS_2_-VA to postoperative stroke and to the occurrence of POAF itself, underscoring that many postsurgical patients cross OAC thresholds if AF is documented. Detection of late POAF often moves patients from “consider” to “warrant” anticoagulation under contemporary recommendations [12].

The principles of long term ECG monitoring offer practical guidance on matching modality to clinical question and time window. Short, intermittent strategies are inherently less sensitive than continuous monitoring; even multiple 24 h Holters miss a sizeable proportion of intermittent AF compared with extended or continuous monitoring. Holters achieve only ~65% sensitivity to detect AF recurrence versus near-continuous approaches, supporting extended windows or hybrid schemes after discharge [13].

Continuous patches (up to ~30 days) improve AF detection in at-risk groups and, in cardiac-surgery populations, increase 30-day detection versus usual care (SEARCH-AF CardioLink trials and related RCTs). These data support continuous or near-continuous strategies in the first month after discharge [14].

Intermittent, patient-activated devices capture symptom–rhythm correlation and extend surveillance economically beyond the initial continuous window and is particularly suited to events occurring after day 10 in our cohort. High-risk ambulatory screening studies (mSToPS) show that patch-first or patch-plus-intermittent strategies increase AF diagnoses and anticoagulation starts, though they may also raise resource utilization, which is relevant when designing pragmatic postsurgery pathways [15]. Our study demonstrates that a substantial proportion of late POAF episodes would be missed with shorter durations of continuous monitoring, underscoring the incremental value of a 30-day patient-activated monitoring strategy. The use of an event recorder, combining scheduled daily recordings with symptom-triggered transmissions, enables capture of the majority of clinically relevant episodes in this population, in whom AF is frequently symptomatic and of sufficient duration to be detected during routine daily measurements.

Implantable loop recorders (ILRs) maximize sensitivity over years but did not reduce stroke versus usual care in the LOOP trial, despite tripling AF detection, highlighting that downstream management and AF burden thresholds matter as much as detection. This is a caution against defaulting to invasive long-term devices purely for screening in unselected postsurgical patients [16].

Consumer wearables/PPG (e.g., Apple Heart Study) scale detection but require confirmatory ECG and careful pathways to avoid over referral. In postsurgical contexts, their role is adjunctive rather than primary, where ECG-confirmed AF is needed to guide OAC [17]. This, however, is feasible with the modern wearable devices available on the market, which allow recording a single lead ECG after a PPG alert of irregular/high heart rate. The wearables, however, as patient-owned devices, are a strategy difficult to apply in a selected population.

Taken together—and in line with our data—the most defensible strategy after CABG/SAVR is continuous or near-continuous ECG in the early phase (to quantify early burden and brady/AV-conduction issues), combined with at least intermittent, patient-activated ECG through day 30 (to capture later, intermittent events). Where systems permit, a full 30-day continuous patch offers the highest detection, but even without the continuous surveillance, the intermittent 30-day monitoring still harvests a substantial yield.

### 4.1. Clinical and Economic Implications

Compared with a 10-day Holter patch, the 30-day patient monitor (PM) strategy increased late POAF detection while reducing per-patient costs (490 vs. 585 PLN), resulting in economic dominance and a negative ICER (−605 PLN per additional AF case; −864 PLN per OAC-eligible case). At an annual volume of ~620 CABG/SAVR patients, PM adoption would yield ~97 additional AF detections and ~68 additional OAC initiations, with an estimated annual saving of ~58,900 PLN.

A structured, 30-day postdischarge monitoring plan can therefore change management in a non-trivial fraction of patients and should be considered part of routine care following CABG/SAVR [2]. In this small, hypothesis-generating study, our findings suggest that a substantial proportion of postcardiac surgery patients may benefit from ECG surveillance using a modality that is cost-effective, has a high detection rate, and is acceptable for patients. Event ECG monitoring appears feasible in this setting, as atrial fibrillation episodes were frequently symptomatic, associated with high ventricular rates, and of sufficient duration to be detected with a high probability. Adding a telemetry feature to the extended event ECG monitoring may allow for even more prompt reaction to avoid potentially fatal complications.

### 4.2. Study Limitations

First, the relatively small sample size (*n* = 55) limits statistical power and precludes definitive conclusions. Given the number of events relative to the number of tested predictors, multivariable models in our study are prone to overfitting, and the associations identified should be viewed as exploratory and hypothesis generating rather than confirmatory evidence. The findings require validation in larger, adequately powered studies before clinical or health-economic inferences can be made.

## 5. Conclusions

Postdischarge POAF is a common and clinically relevant condition. Although it is more frequent among patients with early POAF, it also occurs in a substantial proportion of patients without periprocedural AF, effectively doubling the number of AF diagnoses within 30 days. Although continuous telemetry monitoring would be the option of choice, a pragmatic approach with the use of a patient-activated ECG recorders may represent a feasible and potentially effective approach for postcardiac surgery ECG surveillance. A large-scale application of event ECG, preferably with a telemetry feature, could facilitate timely intervention and potentially prevent severe or even fatal complications. Larger, adequately powered studies are needed to confirm its diagnostic performance, clinical impact, and cost-effectiveness in this population.

## Figures and Tables

**Table 1:** Baseline characteristics of the study population (*N* = 55).

Characteristic	Overall
Age, years	64.6 ± 9.8 (median 67)
Female sex, *n* (%)	21 (38.2)
Hypertension, *n* (%)	36 (65.5)
Diabetes mellitus, *n* (%)	13 (23.6)
Prior myocardial infarction, *n* (%)	12 (21.8)
Prior stroke/TIA, *n* (%)	0 (0)
CHA_2_DS_2_-VA, median (IQR)	2 (0–3)
HAS-BLED, median (IQR)	1 (0–1)
B-blockers prior to procedure, *n* (%)	46 (84)
LVEF, %—median (IQR)	55 (50–60)
Left atrial diameter, mm—median (IQR)	41 (38–44)
Left atrial volume, mL—median (IQR)	23 (22–27)
Procedure, *n* (%)	
CABG	30 (54.5)
SAVR	25 (45.5)
SAVR prosthesis: bioprosthetic, *n*/*N* (%)	19/25 (76.0)
CABG distal anastomoses 2–3, *n*/*N* (%)	20/21 (95.2)

**Table 2:** Postdischarge monitoring metrics.

Measure	*N*	Median (IQR)
Holter patch duration, days	51	9.0 (8.0–10.9)
Event recorder—number of recordings	55	39 (22.0–49.8)
Event recorder—days monitored	55	21 (13.3–28.0)

**Table 3:** Differences in clinical parameters and rhythm disturbances by late POAF status.

Parameter	AF (*n* = 21)	No AF (*n* = 34)	*p* Value
Age, years, median (95% CI)	67.0 (63.0–72.0)	67.5 (63.5–69.0)	0.579
Female sex, *n* (%)	10 (47.6%)	9 (26.5%)	0.147
Hypertension, *n* (%)	14 (66.7%)	22 (64.7%)	1.000
Diabetes, *n* (%)	4 (19.0%)	9 (26.5%)	0.745
* PM sinus tachycardia ≥ 100 bpm, *n* (%)	3 (14.3%)	5 (14.7%)	1.000

* PM sinus tachycardia defined as max heart rate on patient monitor ≥ 100 bpm (proxy for sinus tachycardia episodes on intermittent recordings).

## Data Availability

The data underlying this article will be shared on request to the corresponding author.
